# Impact of lactoferrin supplementation on cotrimoxazole pharmacokinetics: A preliminary clinical investigation

**DOI:** 10.5599/admet.2358

**Published:** 2024-06-27

**Authors:** Dion Notario, Angela Marietha Munzir, Yulina Novella, Linawati Hananta

**Affiliations:** 1 Department of Pharmacy, School of Medicine and Health Sciences, Atma Jaya Catholic University of Indonesia; 2 Department of Pharmacology, School of Medicine and Health Sciences, Atma Jaya Catholic University of Indonesia

**Keywords:** Population pharmacokinetics, urinary excretion, one-compartment, drug interaction

## Abstract

**Background and purpose:**

Cotrimoxazole, a commonly prescribed antibiotic, has substantial resistance, especially in Indonesia, with its uropathogenic resistance reaching 67% in 2017. Although cotrimoxazole has been suggested to be co-administered with lactoferrin to enhance its antibacterial effectiveness and this practice has been widely adopted since the Covid-19 pandemic, the impact of lactoferrin on the pharmacokinetics of cotrimoxazole remains relatively unknown. This study aims to conduct a preliminary clinical investigation into the impact of lactoferrin supplementation on the pharmacokinetics of cotrimoxazole, focusing on the elimination rate and excretion of unchanged drug in urine.

**Experimental approach:**

This study employed a blinded, cross-over, single-dose pharmacokinetics investigation, which included five healthy volunteers as participants. In the initial period, the first group received cotrimoxazole (80 mg trimethoprim and 400 mg sulfamethoxazole) along with a lactoferrin-containing supplement, while the second group only received cotrimoxazole. Subsequently, after a washout period, the conditions were reversed. Urine sampling was conducted at intervals from 0 to 24 hours post-medication, and drug levels in the urine were determined using high-performance liquid chromatography.

**Key results:**

The population-based pharmacokinetic analysis revealed that the optimal model was the one-compartment model with first-order elimination and proportional residual error.

**Conclusion:**

The findings show that the administration of lactoferrin-containing supplements did not significantly influence the covariate model and, therefore, did not alter the pharmacokinetics parameter of cotrimoxazole in urine with a single administration, implying that lactoferrin did not cause drug interaction problems when given simultaneously.

## Introduction

A combination of trimethoprim and sulfamethoxazole (cotrimoxazole) is a widely used antibiotic for treating and prophylaxis infectious diseases, such as bone and joint infections, tuberculosis, and urinary tract infections [1-3]. It is considered generally safe for pediatric, adult, and pregnant patients [4,5], but there is a significant occurrence of resistance to cotrimoxazole in numerous countries [6], including Indonesia, where the uropathogenic resistance to cotrimoxazole was reported to reach 67 % [7].

Various measures are necessary to address antibiotic resistance, including co-administration with lactoferrin [8]. Lactoferrin, a peptide linked to iron ions, aids the body in defending against naturally occurring bacteria and viruses found in milk [9]. It is currently accessible as a dietary supplement in the commercial market, offering beneficial antibacterial outcomes [10,11]. Lactoferrin supplements are seldom sold as individual doses; instead, they are commonly crafted in combination with other ingredients such as colostrum, Echinacea angustifolia, vitamin C, and zinc [12]. Following the onset of the Covid-19 pandemic, there has been a rise in the utilization of supplements that incorporate lactoferrin [13], which has resulted in a growing probability of cotrimoxazole being taken together with lactoferrin-containing supplements.

At present, the interplay between cotrimoxazole and lactoferrin has not been adequately explored. What has been known is that lactoferrin is recognized for its ability to reduce the manifestation of P-glycoprotein (P-gp), a type of efflux transporter [14], while trimethoprim—one component of cotrimoxazole—can function as a substrate for P-gp, indicating that a reduction in P-gp expression can potentially enhance the absorption of trimethoprim within the gastrointestinal tract [15]. Nevertheless, there is no prior documentation of the potential interaction between lactoferrin and sulfamethoxazole, the other component of cotrimoxazole.

To address this gap, this study aimed to explore how supplements containing lactoferrin affect the pharmacokinetics of cotrimoxazole by investigating whether there is a discernible impact associated with the use of lactoferrin-containing supplements on the pharmacokinetic aspects of cotrimoxazole urinary excretion. Specifically, this study focused on assessing the elimination rate and the proportion of unchanged drug excreted in urine, which were both crucial in estimating the cotrimoxazole concentration in the urine for combating uropathogens. As this was a preliminary investigation, assessments were conducted using non-invasive urine samples.

## Experimental

### Pharmacokinetics study

The Ethics Commission of Atma Jaya Catholic University of Indonesia has approved the pharmacokinetics test protocol (01/02/KEP-FKIKUAJ/2023). Prior to the study, five healthy male volunteers underwent a ten-hour fasting period, during which they were only permitted to consume water. The study was conducted in two phases, separated by a seven-day interval. In the initial phase, three volunteers were administered cotrimoxazole, while the other two received cotrimoxazole along with supplements. This arrangement was reversed in the second phase, following a cross-over design. Urine sampling was carried out at intervals of 0-0.5, 0.5-1, 1-1.5, 1.5-2, 2-4, 4-6, 6-8, 8-12 and 12-24 hours post-drug administration. Subsequently, the urine samples were preserved at -20 °C until analysis.

### Sample preparation

Initially, 2.0 mL of the urine sample was transferred into a 10 mL Eppendorf tube. The solution underwent acidification with 1.0 mL of 30 mM sulfuric acid, which was then mixed with 2.0 mL of ethyl acetate and vortexed for 30 seconds. The next stage of separating aqueous and ethyl acetate phases was completed through centrifugation at 3000 rpm for 5 minutes, with the upper ethyl acetate layer isolated and designated as extract A. Meanwhile, the aqueous phase underwent alkalization with 2.0 mL of 80 mM sodium hydroxide solution, and the resulting mixture was partitioned again with fresh ethyl acetate, after which the top ethyl acetate layer was separated and identified as extract B. Both extracts were subjected to evaporation at 85±1 °C using a water bath until dry. The resulting residue was dissolved in 1.0 mL of methanol through ultrasonication for 15 minutes, followed by filtration using a 0.22 μm nitrocellulose filter. The prepared solution was finally transferred to an autosampler vial before being injected into the chromatographic system.

### Chromatographic conditions

The determination of trimethoprim and sulfamethoxazole levels was performed using a Shimadzu LC-20AD high-performance liquid chromatography (HPLC) system equipped with a pump (LC-20AD), autosampler (SIL-20ACHT), oven (CTO-20AC), and PDA detector (SPD-M20A 230V), while the separation was carried out on a Shim-pack GIST®C18 column (150×4.6 mm, 5 μm). For the determination of trimethoprim levels (extract B), the mobile phase consisted of water adjusted to pH 2.5 ± 0.1 with glacial acetic acid and 0.1 M NaOH:acetonitrile (87:13 v/v) and a flowing rate of 1.4 mL/min through the column, which was temperature controlled at 35±1 °C [16]. By comparison, in the sulfamethoxazole determination (extract A), the mobile phase consisted of water adjusted to pH 2.5:methanol:acetonitrile (70:25:5 v/v/v) and a flowing rate of 0.8 mL/min through the column, which was temperature-controlled at 30±1 °C [17].

### Data analysis

**Figure 1.**
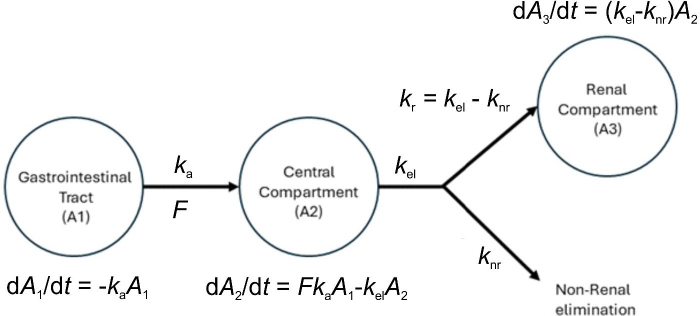
The compartmental model for trimethoprim and sulfamethoxazole using data from urine excretion. *k*_a_ = absorption rate constant, *F* = bioavailability, *k*_el_ = total elimination rate constant, *k*_r_ = renal elimination rate constant, *k*_nr_ = non-renal rate elimination constant, d*A*_1_/d*t* = rate of change of the amount of drug in the gastrointestinal tract; d*A*_2_/d*t* = rate of change of the amount of drug in the central compartment, and d*A*_3_/d*t* = rate of change of the amount of drug in the renal compartment.

The data analysis was conducted using the nlmixr2 package with the R software version 4.3.1 on Ubuntu 22.04.02 LTS 64-bit [18]. The structural model was obtained from a one-compartment pharmacokinetic model featuring first-order kinetics (Figure 1). The dependent variable in this model was the cumulative amount of drug excreted unchanged in urine, while the independent variable was the median of the interval sampling time [19]. The optimal residual model was chosen from a selection of proportional, constant, and combined models. The impact of the supplementation was analysed using a categorical covariate model, with the consideration of the presence or absence of supplementation. The significance of the covariate was determined by observing an improvement in the model when the covariate was added, as evaluated through BIC (Bayesian Information Criterion) and shrinkage. Population parameters were obtained from the best model after bootstrapping.

## Results and discussion

The laboratory tests showed that the participants in the study were in good health, with the five individuals exhibiting comparable age ranges, weights, and heights (Table 1). In this study, a cross-over experimental design, rather than a parallel design, was used to control the effect of biological variation on drug pharmacokinetics more effectively.

**Table 1.** The demographic and laboratory test outcomes of five young male volunteers

Parameters	Mean±SD
Age, year	21.33±1.10
Height, cm	171.33±3.96
Weight, kg	85.67±17.16
Hemoglobin, g / dL	15.63±0.85
Hematocrit, %	48.62±3.31
White blood cells (leukocyte), 10^3^ / μL	6.62±1.39
Red blood cells (erythrocyte), 10^6^ / μL	5.46±0.16
Platelet, 10^3^/μL	300.50±49.32
Mean corpuscular volume, fL	89.02±4.12
Mean corpusculat hemoglobin, pg / dL	28.75±0.90
Mean corpuscular hemoglobin concentration, g / dL	32.25±0.74
Blood urea nitrogen, mg / dL)	17.75±8.22
e-Glomerular filtration rate, (mL / min) / 1.73 m^2^	120.56±14.74
Kreatinin, mg / dL	2.17±3.56
Serum glutamic oxaloacetic transaminase, U / L	20.80±1.04
Serum glutamic pyruvic transaminase, U / L	32.85±8.37

Several pharmacokinetic models with and without covariates in the form of supplementation were compared. However, based on the goodness-of-fit parameters (*i.e.*, BIC and shrinkage), the best model was the one without covariates based on the goodness-of-fit parameters (Table 2), which indicates that short-term administration of a supplement product containing lactoferrin does not affect the pharmacokinetic parameters of either trimethoprim or sulfamethoxazole. The final pharmacokinetic parameters are presented in Table 3.

**Table 2.** Summary of model selection.

Drug	Model	Description	BIC	ΔBIC	Shrink, %		
					*k*_a_	*k*_e_	*k*_nr_
Trimethoprim	T0* (base)	1-compartment, 1^st^ order absorption and elimination, without covariate, estimation method : focei	265.5	0	25.2	-1.48	29.7
	T1	T0 with supplement effect on absorption constant (*k_a_*)	269.8	4.3	24.1	-1.33	29.8
	T2	T0 with supplement effect on total elimination constant (*k*_e_)	1623.5	1358	58.5	3.42	32.9
	T3	T0 with supplement effect on non-renal elimination constant (*k*_nr_)	270.1	4.6	25.3	-1.38	29.6
Sulfamethoxazole	S0* (base)	1-compartment, 1^st^ order absorption and elimination, without covariate, estimation method : saem	694.1	0	20.3	21.7	37.0
	S1	S0 with supplement effect on absorption constant (*k*_a_)	675.8	-18.3	22.2	80.2	2.34
	S2	S0 with supplement effect on total elimination constant (*k*_e_)	685.7	-8.4	19.7	89.8	0.79
	S3	S0 with supplement effect on non-renal elimination constant (*k*_nr_)	679.9	-14.2	20.5	81.1	2.40

*The chosen models for trimethoprim and sulfamethoxazole are T0 and S0, respectively

**Table 3.** Pharmacokinetic characteristics of trimethoprim and sulfamethoxazole determined using the chosen model

Drug	Parameter	Population parameters (95 % CI)	Between subject variability, %
Trimethoprim	*k*_a /_ h^-1^	17.1 (1.83 – 161)	176
	*k*_e_ / h^-1^	0.113 (0.077 – 0.167)	96.8
	*k*_nr_ / 10^-4^ h^-1^	3.13 (1.28 – 7.65)	85.9
	Residual error, mg	0.294	-
Sulfamethoxazole	*k*_a_ / h^-1^	4.95 (1.58 – 15.5)	111.0
	*k*_e_ / h^-1^	0.298 (0.257 – 0.345)	5.40
	*k*_nr_ / h^-1^	0.252 (0.211 – 0.303)	3.91
	Residual error, mg	0.417	

*k*_a_: first-order absorption rate constant, *k*_e_: first-order total elimination rate constant, *k*_nr_: first-order non-renal elimination rate constant

**Figure 2.**
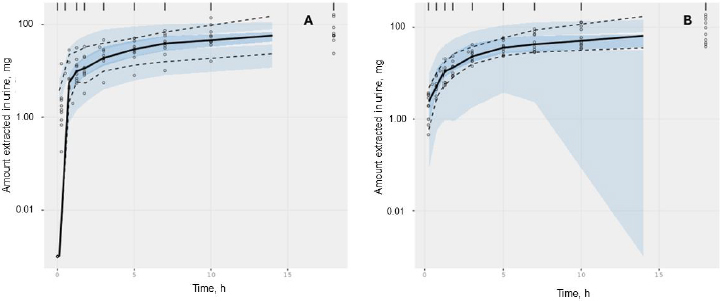
Plot visual predictive check of trimethoprim (A) and sulfamethoxazole (B) from the chosen model. The bold black line depicts the median quantity of drug excreted in urine, while the semitransparent steel blue area represents the simulation-derived 95 % confidence interval around that median. Additionally, the observed 5^th^ and 95^th^ percentiles are indicated by dashed black lines, and the corresponding model-predicted percentiles are visualized as semitransparent sky blue regions.

**Figure 3.**
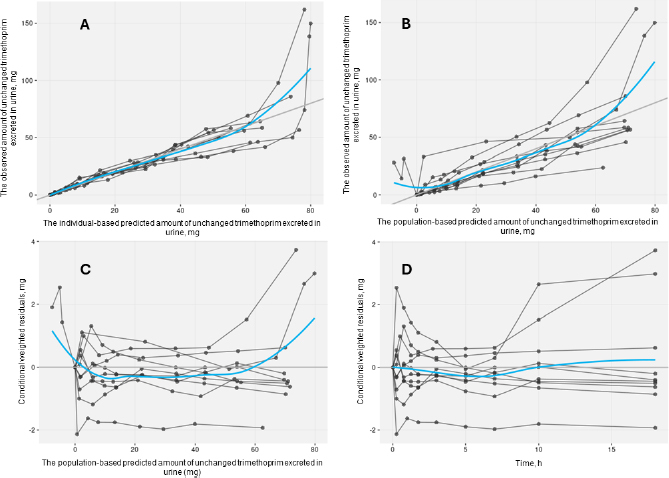
Goodness-of-fit plots of the selected trimethoprim model without covariate. Black points represent individual data observations. Blue lines depict the loess smoothing of the data. Black lines correspond to lines of identity.

**Figure 4.**
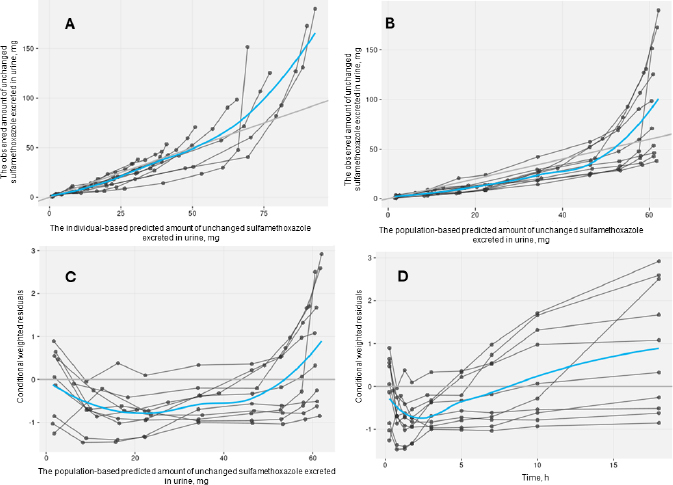
Goodness-of-fit plots of the selected sulfamethoxazole model without covariate. Black points represent individual data observations. Blue lines depict the loess smoothing of the data. Black lines correspond to lines of identity.

The acceptable goodness-of-fit of the final models of trimethoprim and sulfamethoxazole was also assessed. The visual predictive check revealed that the final population of the pharmacokinetic model for trimethoprim and sulfamethoxazole demonstrated effective predictive performance, with the simulation-based prediction interval and the 95 % confidence interval appropriately encompassing the observed median, reaching the 2.5^th^ and 97.5^th^ percentiles of the data (Figure 2A-B). In the comparison between predicted and observed drug amounts in urine, a symmetrical distribution of data points was evident around the regression line (Figure 3A-B and 4A-B). The graph depicting conditional weighted residuals (CWRES) against predictions and time exhibited a random dispersion of data points around CWRES = 0, with the majority of residuals falling within the range from -2 to 2 (Figure 3C-D and Figure 4C-D).

As far as the current literature suggests, this study is the first attempt to analyse the interaction between lactoferrin-containing supplements and the antibiotic cotrimoxazole. It has been discovered that the pharmacokinetics of trimethoprim and sulfamethoxazole can effectively be modelled using a one-compartment model with first-order elimination and proportional residual error, as well as without covariates. On one hand, this aligns with the earlier assumption that the pharmacokinetic parameters of sulfamethoxazole remain unaffected by supplement administration. However, the findings contradict our hypothesis that the absorption of trimethoprim would be influenced by supplement intake.

The anticipated enhancement in trimethoprim absorption in the presence of P-gp inhibiting lactoferrin did not meet the study’s prediction. The lack of change in trimethoprim’s absorption rate may be attributed to the brief supplementation period, which was insufficient for the downregulation process of P-gp [14]. In this study, the supplementation was administered only once, concurrently with the drug, which might be the primary reason the absorption rate of trimethoprim did not change; nevertheless, further investigation is needed.

As identified in this study, the absorption constant values (*k*_a_) for trimethoprim and sulfamethoxazole align with those reported in previous research [20,21]. Additionally, the total elimination constant (*k*_e_) values were validated by indirectly comparing them with clearance (*CL*) divided by apparent distribution volume (*V*) within the established range [22]. Our findings indicate that the *k*_e_ values for trimethoprim and sulfamethoxazole in this study align closely to or fall within the ranges reported in prior studies [21,22]. Notably, the non-renal elimination rate constant (*k*_nr_), which relatively lacks prior publication in pharmacokinetic studies, exhibited a minimal and potentially clinically insignificant value for trimethoprim but a relatively high value that approaches the *k*_e_ value for sulfamethoxazole. This discrepancy stems from the substantially higher urinary metabolism of sulfamethoxazole, which amounts to approximately 70 %, compared to the liver metabolism of trimethoprim, which ranges from 10-30 % [23].

To summarize, the administration of supplements containing lactoferrin demonstrated no noteworthy influence on the pharmacokinetic profiles of cotrimoxazole in a cohort of healthy volunteers. As such, no specific modifications to dosage are deemed essential. Consequently, the concomitant use of lactoferrin supplements and cotrimoxazole can be confidently encouraged without any concern for potential pharmacokinetic interactions.

## Conclusions

Based on the pharmacokinetic modeling results using urine excretion data, no significant impact of administering supplements containing lactoferrin on the pharmacokinetic parameters of cotrimoxazole (trimethoprim and sulfamethoxazole) was observed in the single-dose administration.
